# A proof of principle for using adaptive testing in routine Outcome Monitoring: the efficiency of the Mood and Anxiety Symptoms Questionnaire -Anhedonic Depression CAT

**DOI:** 10.1186/1471-2288-12-4

**Published:** 2012-01-10

**Authors:** Niels Smits, Frans G Zitman, Pim Cuijpers, Margien E den Hollander-Gijsman, Ingrid VE Carlier

**Affiliations:** 1 Department of Clinical Psychology, Faculty of Psychology and Education, VU University, Amsterdam, The Netherlands; 2 Department of Psychiatry, Leiden University Medical Center, Leiden, The Netherlands; 3 Mental Health Institution Rivierduinen, Leiden, The Netherlands

## Abstract

**Background:**

In Routine Outcome Monitoring (ROM) there is a high demand for short assessments. Computerized Adaptive Testing (CAT) is a promising method for efficient assessment. In this article, the efficiency of a CAT version of the Mood and Anxiety Symptom Questionnaire, - Anhedonic Depression scale (MASQ-AD) for use in ROM was scrutinized in a simulation study.

**Methods:**

The responses of a large sample of patients (*N *= 3,597) obtained through ROM were used. The psychometric evaluation showed that the items met the requirements for CAT. In the simulations, CATs with several measurement precision requirements were run on the item responses as if they had been collected adaptively.

**Results:**

CATs employing only a small number of items gave results which, both in terms of depression measurement and criterion validity, were only marginally different from the results of a full MASQ-AD assessment.

**Conclusions:**

It was concluded that CAT improved the efficiency of the MASQ-AD questionnaire very much. The strengths and limitations of the application of CAT in ROM are discussed.

## Background

In the clinical field, self report questionnaires are frequently used to assess mental health, and there is a high demand for efficient assessments [1-3]. This demand is most apparent in mental health institutes which are involved in Routine Outcome Monitoring (ROM, [4,5]), a method devised to collect data on the effectiveness of treatments in clinical practice. It involves recording the outcome of treatments through repeated assessments, which allow for monitoring the development of patient characteristics through time. In this setting, care providers and patients only have a very limited amount of time, and assessments should be short.

A very successful methodology on this point is Computerized Adaptive Testing (CAT). CAT involves the administration of a test or questionnaire via the computer, and at its core lie psychometric models. Each item is dynamically selected from a pool of items and is most informative for the responder in question. The CAT stops when a given level of measurement precision is attained, and typically needs substantially less items than the full item set. In the last decade, CAT has received a lot of attention in the field of quality of life research. For example, the Patient Reported Outcomes Measurement Information System (PROMIS [2]) project has as its goal the development of CATs for the measurement of physical and mental outcomes which allow for monitoring the health-related quality of life of medical patients. CATs have now been developed for depression [6,7] and anxiety [8].

Although these CATs are readily available, there are at least two reasons for clinicians and researchers not using them in ROM. In the first place, a CAT may not have been developed in the right language. For example, Dutch researchers wishing to implement the Depression-CAT [6] need to first translate its items from German into Dutch and then validate them in a relevant Dutch sample, which may simply be too much of an effort. Likewise, clinicians may be used to assessing mental health with an existing ROM instrument with familiar content, and therefore switching to an alternative measure may be unappealing. Instead of switching to existing CATs it may be feasible to convert the familiar scale into a computer-adaptive version. If clinics have collected large samples of item scores of their preferred scale using ROM, they can use these data to validate its items under a psychometric model, and use the outcomes to develop a new CAT, allowing for much shorter future assessments.

The present paper has two goals. The first is to show that the efficiency of the assessment of the Mood and Anxiety Symptom Questionnaire - Anhedonic Depression scale (MASQ-AD [9]) can be substantially improved by building an adaptive version of it. Although several studies on building CAT versions of existing clinical scales have been conducted [1,10], these studies focused on research settings, and not on the assessment of mental health patients. Our second goal is therefore to show that such CATs are an efficient method for monitoring patients in clinical settings as well. We used the ROM-data of a large sample of Dutch patients who filled out the full MASQ-AD as input for a CAT simulation: for each respondent, the actual responses of the full administration were treated as if they had been collected adaptively.

This article has the following structure. In the methods and results sections, the CAT simulation and its outcomes are described. To ameliorate readability, the evaluation of the psychometric quality of the MASQ-AD, which is a prerequisite for applying CAT but -obviously- not the main topic of this study, is presented in the Appendix. Finally, we discuss some benefits and limitations of the application of this methodology in ROM and mental health research in general.

## Methods

### Participants

The sample consisted of 3,597 patients (63% females) from three outpatient centres of Psychiatric Regional Mental Health Care Centers Rivierduinen with an average age of 38.79 years (*SD *= 13.22, range 17-91). Patients were referred to these clinics by their general practitioner for a potential mood, anxiety or somatoform disorder. The diagnosis was assessed with a standardized diagnostic interview, the Mini International Neuropsychiatric Interview (MINI-plus [11]), which was carried out by a research nurse (a psychiatric nurse or a psychologist). According to the MINI, 46% of the patients suffered from depression (4% minor, 42% major depression), 43% suffered from an anxiety disorder, and 17% suffered from a somatoform disorder. In addition, comorbidity among the disorders was found: 2% of the patients had all three disorders, 3% had both anxiety and somatoform disorder, 3% had both depression and somatoform disorder, and 18% suffered from both depression and anxiety; 8, 20, and 23% exclusively suffered from somatoform, anxiety and depression disorder, respectively, whereas 23% had no disorder.

Rivierduinen collaborates with the Department of Psychiatry of the Leiden University Medical Center (LUMC) in the development of ROM. At intake, patients were informed that ROM is part of the general policy of Rivierduinen and LUMC to monitor treatment outcome, that outcomes are made available only to their therapist, and that the data would be used for research purposes in anonymous form. If patients objected to such use, their data would be removed. A comprehensive protocol safeguarded anonymity of the patients and ensured proper handling of the data. This protocol (Psychiatric Academic Registration Leiden database) was available for patients on request, and informed consent was not required. The Medical Ethical Committee of the LUMC approved the regulations and agreed with this protocol (for more details, see De Beurs et al. [4]).

### The MASQ

The MASQ [9,12] is a 90-item self-report questionnaire which has three scales: Anhedonic Depression (AD), Anxious Arousal (AA) and General Distress (GD). AD measures (the lack of) positive affect, AA measures symptoms of somatic arousal and GD measures non-specific symptoms for a depressive or an anxiety disorder. The responder is asked to indicate on a Likert scale (0 = not at all, 2 = a bit, 3 = moderately, 4 = much, 5 = very much) how much they have felt or experienced these stated feelings or thoughts in the past week including today. The Dutch adaptation of the MASQ [13] was used. Here, we focus on the development of a CAT for the AD sub-scale, which contains 22 items. The content of the items is presented in the left part of Table 1.

**Table 1:** Estimated GRM parameters of the items of the MASQ-AD (*N *= 3597).

Nr. (MASQ Nr.)	Item	Item Parameter Estimates				
		
		*a*	*b*_1_	*b*_2_	*b*_3_	*b*_4_
1 (1)	Felt cheerful (+)	2.41	-2.50	-1.08	-0.08	0.78
2 (14)	Felt really happy (+)	2.61	-2.52	-1.44	-0.57	0.18
3 (18)	Felt optimistic (+)	2.37	-2.74	-1.47	-0.60	0.40
4 (21)	Felt really bored (-)	0.98	-0.40	0.81	1.86	3.58
5 (23)	Felt like I was having a lot of fun (+)	2.75	-2.77	-1.51	-0.69	0.08
6 (26)	Felt withdrawn from other people (-)	1.18	-0.46	0.74	1.44	2.81
7 (27)	Seemed to move quickly and easily (+)	1.41	-3.05	-1.33	-0.26	0.68
8 (30)	Looked forward to things with enjoyment (+)	2.55	-2.11	-0.98	-0.25	0.61
9 (33)	Felt like nothing was very enjoyable (-)	2.08	-0.59	0.26	0.80	1.77
10 (35)	Felt like I had accomplished a lot (+)	1.74	-3.49	-2.03	-1.07	-0.20
11 (36)	Felt like I had a lot of interesting things to do (+)	1.94	-3.34	-1.86	-0.98	-0.17
12 (39)	Felt like it took extra effort to get started (-)	1.08	-1.72	-0.40	0.34	1.93
13 (40)	Felt like I had a lot to look forward to (+)	2.21	-2.65	-1.43	-0.62	0.27
14 (44)	Felt like there wasn't anything interesting or fun to do (-)	1.38	-0.72	0.33	1.10	2.39
15 (49)	Was proud of myself (+)	2.06	-2.84	-1.68	-0.77	0.29
16 (53)	Felt unattractive (-)	0.90	-0.68	0.57	1.49	2.65
17 (58)	Felt really "up" or lively (+)	2.90	-2.72	-1.65	-0.81	-0.20
18 (66)	Felt really slowed down (-)	1.15	-0.75	0.51	1.34	2.75
19 (72)	Felt like I had a lot of energy (+)	2.01	-3.00	-1.79	-0.84	-0.14
20 (78)	Felt hopeful about the future (+)	1.96	-2.59	-1.40	-0.53	0.44
21 (86)	Felt really good about myself (+)	2.63	-2.88	-1.57	-0.69	0.22
22 (89)	Thought about death or suicide (-)	1.18	0.86	1.71	2.26	3.34

Note. *a *is the discrimination parameter; the *b*'s are threshold parameters.

A plus represents a positively and a minus a negatively formulated item; positively formulated items were score-reversed.

In the Appendix a psychometric evaluation of the MASQ-AD using the current sample is presented. On the basis of this evaluation it was concluded that the item set of the MASQ-AD was a valid input for an adaptive test.

### The Simulated MASQ-AD CAT

A CAT algorithm usually consists of five components [14,15]. The first component consists of the estimated parameters of an appropriate Item Response Theory (IRT) model for the items which form the item 'pool'. The second component is a method to select new items during the assessment; the third is a method to make a (provisional) estimate of the subject's scale score after the administration of each item. Because a provisional score estimate is unavailable before administrating the first item, a starting level is specified, which is the fourth component. The fifth component of CAT is a rule which specifies when to stop the assessment. Details associated with the five CAT components of our simulation, are presented in what follows.

To obtain *item parameters *of the MASQ-AD items, the Graded Response Model (GRM [16]) for polytomous items, was fit to the data. Although other IRT model candidates exist, the GRM is often preferred because (*i*) it has parameters which can be easily interpreted in terms of the responder behavior [17], and (*ii*) it is easier to understand and illustrate to users than the other models [18]. Like all IRT models, the GRM assumes that the responses to the items of a questionnaire are accounted for by a latent construct (often denoted by *θ*) and characteristics of the items. It specifies two types of item parameters which quantify the relationship between the latent trait and the item score. Each item has one discrimination, or *a*, parameter, which expresses the discriminative power of an item to demarcate differences between respondents of similar scores on the latent trait. In addition, it has one or more threshold, or *b*, parameters which specify the location on *θ *on which one is expected to step from a lower to a higher item category; the number of *b*s per item is equal to the number of item categories minus one. A more thorough description of the GRM and the meaning of its parameters can be found in other sources [10,19].

The estimates of the complete sample (see, Table 1) are evidently the best estimates of the population GRM parameters. It would, however, be unfair to use these estimates in the present CAT simulations. Utilizing the same sample to both calibrate the items and to simulate the CAT upon, may lead to overfitting [20], giving outcomes which are too optimistic. To deal with this issue, two-fold cross validation [21] was performed: the sample was randomly split in two equally sized groups. In each of the two sub-samples, the parameters of the GRM were estimated. Next, each set of estimates was used as input for the CAT of respondents in the other sample. In other words, for each subject, the simulated CAT used parameter estimates that were obtained in the sub-sample (s)he did not belong to.

Like in most other CATs, the method of *selecting new items *was based on item information [15,19]. Item information quantifies with how much precision an item can measure the latent trait given the location of the provisional person estimate [22]. In the simulation study, the CATs selected that new item which had the highest information at the provisional estimate of *θ*.

Two *score estimation methods *are available in IRT: Maximum Likelihood (ML) and Bayesian estimation [19]. The ML approach estimates *θ *as that value which has the highest likelihood of bringing forth the responses observed [23]. By contrast, Bayesian estimation uses, in addition to this likelihood, an a priori population distribution of the latent variable, such as the standard normal. Consequently, Bayesian estimation can and ML estimation cannot provide an estimate for item response patterns consisting exclusively of either extreme lower or extreme higher categories. Because such response patterns were anticipated in the present study, the estimation of *θ *was performed using a Bayesian method, called Maximum a Posteriori (MAP [19]), which assumed *θ *to follow a standard normal distribution.

In the CAT procedure the *starting level *was set to the average value of the latent trait, which was 0. As a consequence, the item with the highest information at this initial latent depression value, Item 5, was chosen as the first item for all respondents.

Generally, there are two types of *stopping rules *for terminating a CAT: either a fixed number of items administered or a pre-specified level of measurement precision. The latter criterion, which was used in this study, is met when the subject's Standard Error (SE) of *θ *is small enough. To illustrate the impact of this rule, the CAT was run under several levels of minimally required SEs of *θ *(0.2, 0.3, 0.4, 0.5, and 0.6).

To simulate the adaptive version of the MASQ-AD, a CAT program [10] was written in the statistical environment R [24]. To perform a simulation for a particular questionnaire, the program needs (a) estimates of its GRM parameters and (b) a data file with scores on its items from a sample from the population in question as input. In the simulation, for each responder in the sample, the full set of MASQ-AD item responses was used, and item scores were selected from it and evaluated as if they were being collected adaptively.

### Comparing complete and CAT data

Evidently, for an adaptive MASQ-AD to be efficient, its outcomes should be very similar to those of the full assessment. Moreover, the *usefulness *of the CAT estimate, for example in diagnosing depression, should be similar to that of the fixed questionnaire estimate.

Two analyses were performed to determine the extent to which CAT assessment were in accordance with the full assessment. In the first place, CAT estimates of latent depression were compared with estimates resulting from the full assessment (i.e., 22 item scores), using Pearson correlations between the estimates. The other analysis focused on the correspondence in criterion-related validity [22] between fixed-test and CAT estimates; that is, these two types of estimates were compared at their relation with three other measures. Two criteria were the total scores of the two other sub-scales of the MASQ: AA and GD. The validity was determined using the Pearson correlation coefficient. The third criterion was an 'either major or minor' depression classification based on the MINI diagnosis. The predictive utility for this outcome was expressed in the Area Under the Curve (AUC) of the receiver operating curve, which is an effect size for expressing clinical significance [25]. It was presented here to provide clinicians with a quantification of the impact of CAT on predictive utility. In the present study, AUC may be interpreted as the probability that a randomly selected depressed person scores higher on the MASQ-AD than a randomly selected person without depression [26].

### Software

LISREL [27] was used for the Confirmatory Factor Analysis (CFA, see Appendix). All other analyses (see, Appendix) were performed using several R [24] libraries. Item parameters were estimated using the ltm [28,29] library. The polychoric correlations were obtained with the polycor library [30]. Mokken scaling was performed using the mokken library [31,32]. DIF detection was performed using the lordif library [33,34]. The code of the CAT algorithm comprised an alteration of, and additions to the code of the ltm library, and is obtainable from the first author.

## Results

### Characteristics of the CAT

Table 2 presents several features of the CAT procedure under the different stopping rules. The first row shows, as a reference point, the outcomes of the CAT with no stopping rule. The first and second column show the average and standard deviation (SD) of the number of items administered; the third column presents the percentage of respondents for whom all 22 items had to be administered. Clearly, the higher the required level of measurement precision, the higher the number of items administered.

**Table 2:** Characteristics of the CAT under several stopping rules.

Stopping rule	Number of Items Used			Mean SE(*θ*)	Marginal Reliability	Correlation between CAT *θ *and Complete Test *θ*
				
	Mean	SD	% All			
None	22.000	0.000	100.0	0.231	0.945*^a^*	1.000
SE(*θ*) *<*0.2	19.726	3.504	65.3	0.234	0.944	0.999
SE(*θ*) *<*0.3	8.644	5.712	11.0	0.298	0.910	0.979
SE(*θ*) *<*0.4	4.484	3.849	2.9	0.373	0.850	0.946
SE(*θ*) *<*0.5	2.789	1.840	0.3	0.425	0.793	0.917
SE(*θ*) *<*0.6	1.505	0.520	0.0	0.521	0.577	0.837

*^a ^*Coefficient alpha for the full MASQ-AD was 0.940.

**Figure 1:**
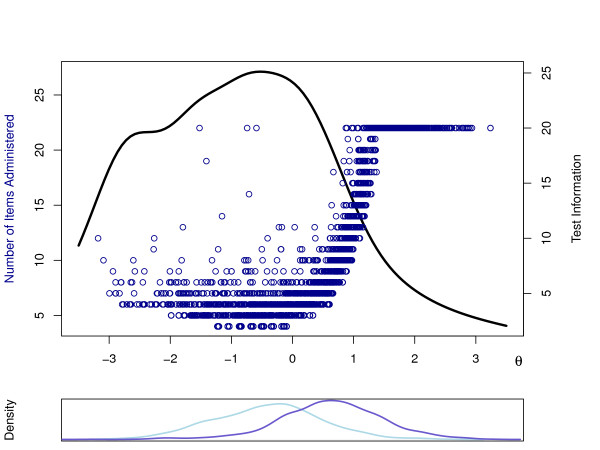
**The upper panel shows the relationship between the latent depression (*θ*) estimate and the number of administered items for stopping rule 'SE(*θ*) *<*0.3' (blue dots represent respondents)**. The black curve represents test information as a function of *θ*. The lower panel shows the kernel density estimates of the distribution of *θ *in the group with (dark blue line) and without (light blue line) a depression diagnosis according to the MINI.

The fourth column shows the average SE of the final estimate of *θ*s for each level of measurement. The average SE under the 'SE(*θ*) *<*0.2' stopping rule is higher than the required SE; obviously, this resulted from the 65.3% of the respondents for whom the item pool was exhausted before the pre-specified measurement was met (i.e., SE was higher than desired). The upper panel of Figure 1 shows the number of items administered as a function of the estimated *θ *in the CAT with the 'SE(*θ*) *<*0.3' stopping rule. From this figure [Figure 1] it becomes clear that the 11% of respondents making all 22 items were mostly found on the right-hand side of the latent depression scale. This is a result of the test information being relatively low in that region (for the three subjects left to the center, the full set of items was needed because of inconsistent response behavior: they chose low categories for easy to endorse items and high categories for difficult to endorse items).

To provide readers unfamiliar with IRT a sense of the meaning of the CAT SEs, we present an estimate of marginal reliability [35] in the fifth column of Table 2. In IRT, there is no single estimate of reliability, because measurement precision varies as a function of the latent trait. Marginal reliability is a rather crude estimate of IRT measurement precision when test information is peaked; therefore it should be kept in mind that the current marginal reliability estimates are inaccurate, and used for illustrative purposes only. Nevertheless, it is obvious that reliability decreases as the CAT uses a stopping rule with a higher standard error.

The sixth column presents the correlations between the complete data and CAT *θ *estimates. These correlations were quite high. For example, whilst the respondents answered, on average, only 5 of the 22 items in the 'SE(*θ*) *<*0.4' CAT, a correlation of 0.946 was found. Self-evidently, the correlations decreased as measurement precision decreased, with a somewhat lower correlation (0.837) when, on average, administering only 1.5 item in the 'SE(*θ*) *<*0.6' CAT.

### Criterion Validity of the CAT

Table 3 shows the relationship between the CAT estimates and the three criterion variables. Results were highly similar for these three measures: the criterion-related validity was an increasing function of the required measurement precision of the CAT.

**Table 3:** Relationship with external criteria (95% confidence intervals between brackets) of the CAT estimates under several stopping rules.

Stopping rule	MASQ-AA(*r*)	MASQ-GD (*r*)	Any Depression (auc)
None: Sum score	0.499 (0.475 - 0.524)	0.784 (0.772 - 0.797)	0.815 (0.801 - 0.829)
None: $\hat{\theta }$	0.460 (0.434 - 0.486)	0.739 (0.724 - 0.754)	0.810 (0.795 - 0.824)
SE(*θ*) *<*0.2	0.453 (0.427 - 0.479)	0.730 (0.715 - 0.746)	0.807 (0.793 - 0.822)
SE(*θ*) *<*0.3	0.414 (0.387 - 0.442)	0.685 (0.668 - 0.703)	0.794 (0.779 - 0.809)
SE(*θ*) *<*0.4	0.396 (0.368 - 0.423)	0.651 (0.632 - 0.670)	0.782 (0.767 - 0.797)
SE(*θ*) *<*0.5	0.385 (0.358 - 0.413)	0.627 (0.607 - 0.647)	0.769 (0.754 - 0.784)
SE(*θ*) *<*0.6	0.336 (0.307 - 0.365)	0.556 (0.533 - 0.578)	0.746 (0.730 - 0.762)

Note. *r *is the Pearson correlation; AUC is the area under the ROC-curve.

The correlation of the full CAT *θ *with the MASQ-AA total score was 0.460. This correlation diminished as measurement requirements went down. For example, when using the 'SE(*θ*) *<*0.5' rule (average number of items administered 2.789), the correlation dropped to 0.385. The correlation of the 'no stopping rule' CAT *θ *and the MASQ-GD total score was 0.739 (column two). This value became smaller as the measurement requirements decreased. For example, the CAT using the 'SE(*θ*) *<*0.6' rule (administering on average only 1.5 item) gave a correlation of 0.556.

The last column of Table 3 reports on the CAT's diagnostic accuracy, expressed in AUC, for the depression classification. The diagnostic accuracy of the estimates of *θ *based on the 'no stopping rule' was high and went down somewhat as measurement requirements decreased. However, the estimates remained clinically significant under all stopping rules: all AUCs were higher than the value commonly used as a lower bound for a large effect [36].

## Discussion and Conclusions

In this study it was shown how CAT can successfully be applied as an efficient method of reducing the length of the administration of the MASQ-AD in ROM. Simulated CATs under five stopping rules (required standard errors in decreasing steps of 0.10) were performed. Naturally, when increasing the required measurement precision, the average number of administered items increased. Likewise, the relationship between the latent depression estimates using the full and adaptive assessment increased as measurement requirements increased. Moreover, with increasing required measurement precision, the criterion validity of the latent depression estimate was decreasingly attenuated by measurement error.

In spite of the obvious loss of information as requirements of measurement precision were relaxed, the magnitude of this loss was surprisingly low. For example, the CAT requiring SE to be at least 0.4, recording on average only about a fifth part of the items per respondent, gave depression scores that correlated 0.946 with the full assessment score, and 0.651 with the MASQ-GD scale score, which was only marginally smaller than the original concurrent validity (0.739). In addition, the CAT estimates under the 'SE(*θ*) *<*0.5' stopping rule, recording on average only about a seventh of the items per respondent, correlated 0.917 with the original score, and had an AUC (0.769) for predicting depression that was only marginally smaller than that of the full assessment estimates (0.810). This study, therefore, links up to other studies on CAT [1,6,7,10] in its conclusion that it is a fruitful way of increasing the efficiency of self report questionnaires.

Before discussing which stopping rule is best for a real MASQ-AD CAT in ROM, it should be stressed that the present outcomes were based on a simulated CAT on data that were obtained in a standard assessment. Obviously, simulated and actual adaptive administrations may yield different results concerning item reductions because respondents may behave differently in reality. Therefore, in addition to the present study, an actual MASQ-AD CAT administration should be studied as well. Fortunately, others have shown that the outcomes of simulated and actual CAT administrations can be very similar [37], which may render the present study instructive nevertheless. Stopping rules 'SE(*θ*) *<*0.4' and 'SE(*θ*) *<*0.5' seem to be the best for real MASQ-AD CAT administrations in patient populations. CATs under these rules used a relatively low number of items, showed a substantive correlation with the original latent depression estimates, and had a small attenuation in criterion-related validity.

In addition to allowing for an improvement of the efficiency of MASQ-AD assessment, IRT modeling provided estimates of test information (see Appendix). This showed that the information peaked somewhat below the middle of the latent trait scale. Figure 1 shows that for *θ >*1, information is relatively low. In addition, the lower panel shows the estimated distribution of the latent depression variable for each of the two MINI groups, separately. It is apparent that the test is informative in the region where these two groups are to be told apart; in the region where the patients suffering from depression prevail (i.e., on the right hand side), information is lower. Consequently, in that region small differences are not as easily detected as for respondents with lower scores of depression (which caused the MINI depression group requiring, on average, about four items more than the no depression group under the 'SE(*θ*) *<*0.3' rule). MASQ users should ask themselves if the test information presented complies with their testing goal. For example, when using CAT for monitoring the development of mental health, and for obtaining reliable change scores, an item bank with more uniform test information may be preferred. By contrast, in other situations MASQ-AD assessment may be much more aimed at deciding whether a respondent scores high on depression or not (see, [38]). In such cases CAT as described in this study seem be a sound choice. Actually, when predictive utility is the only goal, it may be even better to adjust the CAT algorithm and use so called clinical decision adaptive testing [39,40], in which items with threshold parameters around a cut-score are needed. When MASQ-AD users are satisfied with the test information presented in this study, however, they can take advantage of CAT as a method of very efficient MASQ-AD assessment in ROM.

Although CAT provides an opportunity to improve the efficiency in patient assessment substantially, its implementation not only needs knowledge of building an item bank and psychometric analysis. For an actual implementation, a test delivery system is needed, and this system should be flexible enough to incorporate the dynamic nature of CAT. Evidently, the extent of the investment of switching to CAT depends upon the current assessment method. For example, when ROM is performed using web-based software, which is the case at the Rivierduinen Centers, the adaptation does not have to be so extensive. By contrast, for those institutions still using paper and pencil questionnaires, such a system may have to be built from scratch. For such institutions it may be fruitful to register at the Assessment Center (http://www.assessmentcenter.net), a free online research management tool sponsored by PROMIS, which enables mental health workers and researchers to create websites and CATs for their own patients and scales.

In this study we sought to find a solution for the main disadvantage of ROM: its time consumption [5]. Although we restricted our attention to the efficiency of a single scale, the ultimate solution would be to convert all scales used for assessment in ROM to a CAT version. One could add smart testing designs to this [41-43], for example, allowing for the collection of scores for only some time points for each subject. Such a hybrid system would lead to an even larger reduction in time consumption. It should be noted that efficient assessments have, at least, two other advantages. First, for some patient groups, such as severely diseased patients, administering many questions may decrease the quality of the answers given [3]; short assessments will lead to data with higher quality. Second, ROM data can be used by researchers for effectiveness studies [4]. In such studies, it is important that patients who entered treatment remain in treatment. Because the willingness of patients to cooperate in research is known to decreases with the size and number of questionnaires [44,45], shorter ROM assessments may lead to lower drop-out rates. We hope that both mental health researchers and practitioners are convinced by these outcomes and will implement the MASQ-AD CAT or develop a CAT for their preferred mental health instruments for use in ROM.

## Competing interests

The authors declare that they have no competing interests.

## Authors' contributions

NS analyzed the data and prepared the manuscript. FZ, MH, and IC had full access to the ROM data and gave significant comments on the manuscript. PC gave significant comments on the manuscript. All authors have read and approved the final version of the manuscript.

## Appendix

### Psychometric evaluation of the MASQ-AD items

To study the quality of the MASQ-AD items as input of an adaptive version, we followed the methodology as recommended by the PROMIS project [18]. Attention was directed on effect sizes, not on statistical significance. As most model fit statistics are sensitive to sample size, statistically significant outcomes may be trivial (models never fit the data perfectly, and even the tiniest deviations can be detected by increasing sample size).

When building a CAT version of the MASQ-AD, it should be shown that its items comply with several psychometric requirements. CAT relies upon Item Response Theory (IRT), and before applying an IRT model, it is important to evaluate its main assumptions of unidimensionality, local independence, and monotonicity [18]. To study *unidimensionality*, a Confirmatory Factor Analysis (CFA) was performed on the polychoric correlation matrix [46] of the MASQ-AD items. The resulting one factor CFA model had four out of five fit indices (Comparative Fit Index, Tucker Lewis Index, Standardized Root Mean Residuals, and average residual correlations) which showed good fit (for rules of thumb, see [18]); the RMSEA was higher (0.097) than what is commonly required for excellent fit (*<*0.06). In addition, the first factor in a principal components analysis on the polychoric correlations accounted for 54% of the questionnaire variance, more than adequately meeting the Reckase criterion of 20% ([47] cited in [48]). In addition, the second factor only explained 9% of the variance which gave a ratio of variance explained of the first to the second factor of about six, which is higher than the required minimum of four [18]. On the basis of these results we concluded that the MASQ-AD items shared a single common factor.

Under the assumption of *local independence*, there should be no covariance left among the items after controlling for the dominant factor. To test local dependence, the matrix of residual correlations resulting from the one factor CFA was studied; coefficients with values higher than 0.2 were considered as possibly locally dependent [18]. Only one out of the 231 (1*/*2 *× *21 *× *22) item pairs was marked as possibly locally dependent: items 12 ('Felt like it took extra effort to get started') and 18 ('Felt really slowed down') had a value of 0.29. It is apparent that, in addition to being an indicator of depression, both items are associated with the respondent feeling 'sluggish'. By contrast, the modification indices in LISREL [18], were all equal to zero. In addition, local independence under the GRM was studied using Yen's [49] Q3 statistic. This statistic calculates the residual item scores under the GRM (i.e., observed - expected scores), and correlates these among items. To assess lack of model fit, we used Cohen's [50] rules of thumb for correlation effect sizes: Q3's between 0.24 and 0.36 are moderate deviations, and values of 0.37 and larger represent large deviations. Four of the 231 item pairs had Q3 values that showed at least a moderate deviation of model fit: item pairs 9 and 14, 10 and 11, 17 and 19, had moderate values, whereas pair 12 and 18 showed a large value (0.39). The outcomes suggested that there may exist a specific relationship between some items over and above their association with the latent depression score. When considering the content of the items this was confirmed. For example, items 12, 17, 18 and 19 are all statements associated with feeling 'sluggish'. It may be argued that the MASQ-AD has some imbalance in its item design in that some aspects, such as feeling sluggish, appear in more items than others, which causes them to co-vary after controlling for the dominant dimension. Although most of these deviations were only moderate, one item pair (item 12 and 18) showed lack of local independence both under the one factor model and GRM, which would qualify one of the items for possible removal. To study the issue in more detail we followed the recommendations of Reeve et al. [18] and fitted two new GRMs, one after removing item 12, and one after removing item 18 from the item pool. In both cases, fortunately, the parameters of the model hardly changed: the largest change in *b *parameters was six hundredths, and the largest difference in *a *parameters was four hundredths.

Under the *monotonicity *assumption, the probability of choosing a higher item category should increase or remain constant, but should not decrease with increasing values on the dominant dimension. The monotonicity of the MASQ-AD items was studied using Mokken scaling [51], which is based on a non-parametric IRT model, and can easily be engaged to visually inspect estimates of such probabilities. Mokken scaling showed that the MASQ-AD scale complied with monotonicity to a high extent: all 22 items showed monotone increasing functions with increasing sum scores. The accompanying scalability coefficient [51] of the whole scale was 0.49, which according to rules of thumb [31] is a scale of moderate quality. All of the items had a scalability coefficient that was higher than the lower bound of 0.3. On the basis of these outcomes it was concluded that the MASQ-AD items met the assumption of monotonicity.

**Figure 2:**
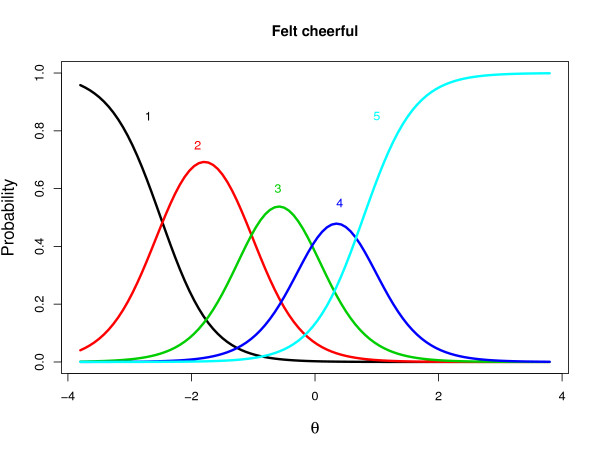
**Estimated category response curves for item 1 of the MASQ-AD scale**. Note that the item was reverse-scored (5 = not at all, 4 = a bit, 3 = moderately, 2 = much, 1 = very much).

After these basic assumptions were confirmed, the GRM was fit to the data. To study the *model fit of GRM *on the MASQ-AD items, several analyses were performed. First, item parameters were estimated and category response curves (CRCs) were plotted per item to see on what intervals on the latent depression scale each of the five item categories were most frequently chosen (see Figure 2, for the CRCs of item 1). The parameter estimates of the GRM for the MASQ-AD items are shown in Table 1. The second column of the table shows the *a *(discrimination) parameters; item 16 has the lowest, and item 17 has the highest strength of association with the latent depression variable. The other columns show the estimates of the threshold parameters. In addition, the item and test information [19] plots showed that the MASQ-AD scale was most informative near the average latent depression score in the current population. The test information (also, see [Figure 1], right-hand vertical axis) was roughly bell-shaped, with a peak at *θ *= *-*0.50, and about 60% of the information for *-*2 *< θ <*1. In addition, on the basis of the estimated item parameters, for each patient a latent depression score (*θ*) was estimated.

Second, we followed the suggestion of Reeve et al. [18] in using the *G*^2 ^statistic [52] to compare observed and expected response frequencies under the estimated IRT model. For a proper use of *G*^2 ^it is necessary that the expected frequencies in the cells are at least 5, however, and its use was problematic because for as much as 13 of the 22 items it was impossible to obtain expected values of at least 5. This was mainly because under the GRM, respondents with high *θ *were almost never expected to choose one of the lower item categories. Consequently, *G*^2 ^was of no use, and therefore for assessing GRM model fit we focused on other outcomes.

Third, another assumption of IRT models is that the item parameters apply for all respondents. If parameters differ between groups, a test is said to suffer from Differential Item Functioning (DIF, [19]). The consequence of DIF is that respondents from different groups, who actually have an identical score on the latent trait, have an unequal probability of endorsing an item [53]. Consequently, the estimated latent trait scores may be different. Adaptive tests may be more vulnerable to the effects of DIF on validity [15,18], because in shorter assessments DIF items may have a higher impact. We performed DIF analysis with respect to age, gender, and diagnostic group using ordinal regression methods [54]. As a measure of effect size we used the change in McFadden's *R*^2^, and followed the suggestion of using a value of 0.02 [33] as a critical value for rejecting the nulhypothesis of no DIF. Fortunately, no DIF items were detected.

As a final step in assessing GRM model fit, the latent depression score was estimated for all respondents, and correlated with the traditional MASQ-AD sum score. The correlation was 0.98, which was interpreted as a good fit of the GRM to the data as well.

The psychometric analyses showed that the items of the MASQ-AD scale had many strong features and only a few weaknesses. The item set showed both unidimensionality and monotonicity and no DIF items were detected. By contrast, two methods for detecting local dependence indicated that there was a substantial residual covariation between one pair of items when controlling for the latent trait score. Fortunately, this dependence had a negligible effect on the parameter estimates of GRM; therefore both items were maintained in the item pool. All in all it was concluded that the item set of the MASQ-AD was a valid input for an adaptive test.

## Pre-publication history

The pre-publication history for this paper can be accessed here:

http://www.biomedcentral.com/1471-2288/12/4/prepub
